# Protective Effects of Astragaloside IV on Uric Acid-Induced Pancreatic *β*-Cell Injury through PI3K/AKT Pathway Activation

**DOI:** 10.1155/2022/2429162

**Published:** 2022-01-10

**Authors:** Zhenhuan Jiang, Gang Wang, Lingling Meng, Yunzhao Tang, Min Yang, Changlin Ni

**Affiliations:** ^1^NHC Key Laboratory of Hormones and Development, Tianjin Key Laboratory of Metabolic Diseases, Tianjin Medical University Chu Hsien-I Memorial Hospital and Tianjin Institute of Endocrinology, Tianjin 300134, China; ^2^Department of Cardiology, Gao'an People's Hospital, Gao'an 330800, China; ^3^Department of Endocrinology and Diabetes, Cangzhou Central Hospital, Cangzhou 061000, China

## Abstract

**Background:**

Elevated uric acid (UA) has been found to damage pancreatic *β*-cell, promote oxidative stress, and cause insulin resistance in type 2 diabetes (T2D). Astragaloside IV (AS-IV), a major active monomer extracted from *Astragalus membranaceus* (Fisch.) Bunge. which belongs to TRIB. Galegeae (Br.) Torrey et Gray, *Papilionaceae*, exhibits various activities in a pathophysiological environment and has been widely employed to treat diseases. However, the effects of AS-IV on UA-induced pancreatic *β*-cell damage need to be investigated and the associating mechanism needs to be elucidated. This study was designed to determine the protective effects and underlying mechanism of AS-IV on UA-induced pancreatic *β*-cell dysfunction in T2D.

**Methods:**

UA-treated Min6 cells were exposed to AS-IV or wortmannin. Thereafter, the 3-(45)-dimethylthiahiazo(-z-y1)-35-di-phenytetrazoliumromide (MTT) assay and flow cytometry were employed to determine the effect of AS-IV on cell proliferation and apoptosis, respectively. Insulin secretion was evaluated using the glucose-stimulated insulin secretion (GSIS) assay. Finally, western blot and quantitative real-time polymerase chain reaction (qRT-PCR) were performed to determine the effect of AS-IV on the phosphatidylinositol 3-kinase (PI3K)/protein kinase B (AKT) pathway in UA-treated cells.

**Results:**

AS-IV had no cytotoxic effects on Min6 cells. UA significantly suppressed Min6 cell growth, promoted cell apoptosis, and enhanced caspase-3 activity; however, AS-IV abolished these effects in a dose-dependent manner. Further, decreased insulin secretion was found in UA-treated Min6 cells compared to control cells, and the production of insulin was enhanced by AS-IV in a dose-dependent manner. AS-IV significantly increased phosphorylated (p)-AKT expression and the ratio of p-AKT/AKT in Min6 cells exposed to UA. No evident change in AKT mRNA level was found in the different groups. However, the effects of AS-IV on UA-stimulated Min6 cells were reversed by 100 nM wortmannin.

**Conclusion:**

Collectively, our data suggest that AS-IV protected pancreatic *β*-cells from UA-treated dysfunction by activating the PI3K/AKT pathway. Such findings suggest that AS-IV may be an efficient natural agent against T2D.

## 1. Introduction

Diabetes mellitus (DM), a frequent chronic disease, is known to have a remarkable burden on public health [1]. According to the US diabetes epidemiological statistics, 8% of people had diabetes in 2011, and 80 million adults were diagnosed with prediabetes [2]. Type 2 diabetes (T2D), a type of DM, is characterized by insulin tolerance and *β*-cell damage [3]. Many reports have revealed that *β*-cell dysfunction will appear when the pancreas cannot excrete adequate insulin [4–6]. Thus, protecting pancreatic *β*-cell functions may be an approach for T2D development and therapy. Based on several reports, uric acid (UA) level is associated with *β*-cell functions, insulin secretion, and the risk of T2D [7–10]. Several studies also revealed that increased UA could abduct insulin resistance and inhibit pancreatic *β*-cell survival and insulin product [11–13]. However, the latent mechanism of UA-induced *β*-cell dysfunction in T2D has not been fully elucidated.

Astragaloside IV (AS-IV) is a bioactive saponin purified from *Astragalus membranaceus* which belongs to TRIB. Galegeae (Br.) Torrey et Gray, *Papilionaceae* [14]. Based on accumulating evidence, AS-IV exerts multipotent activities in many diseases, such as anti-inflammation [15] and antioxidant [16], with no toxicity. Zhang et al. revealed that AS-IV extends the lifespan of *Caenorhabditis elegans* by promoting age-related functional declines and inducing antioxidant responses [17]. Some of these pharmacological activities resulted in the regulation of the phosphatidylinositol 3-kinase (PI3K)/protein kinase B (AKT) pathway signaling pathways [18–20]. The PI3K/AKT pathway has been found to be involved in the prevention of UA-induced dysfunction of *β*-cells [8]. For instance, Zhang and Qui revealed that the PI3K/AKT inhibitor LY294002 promotes UA-induced dysfunction of *β*-cells [21]. We speculated that the activation of PI3K/AKT may be involved in the protective effect of AS-IV on UA-induced *β*-cell injury in diabetes.

The aim of this study was to illustrate the potential therapeutic effects of AS-IV on UA-stimulated *β*-cell injury and elucidate the underlying molecular mechanism of AS-IV in T2D.

## 2. Materials and Methods

### 2.1. Cell Culture

The mouse insulinoma Min6 cells were obtained from Shanghai Guandao Biological Engineering Co., Ltd. (Shanghai, China) and grown in Dulbecco's modified Eagle's medium (DMEM; HyClone, USA) supplemented with 10% fetal bovine serum (FBS; Sigma-Aldrich, St. Louis, MO, USA), 100 U/ml penicillin, and 100 U/ml streptomycin (Gibco, Grand Island, NY, USA). The cells were grown in a 5% CO_2_ incubator at 37°C.

### 2.2. Cell Treatment

Min6 cells were treated with UA (5 mg/dl; Sigma-Aldrich, St. Louis, MO, USA) at 37°C for 24 h to induce cell injury. To study the effects of AS-IV (ChengDu Conbon Biotech Co., LTD, Chengdu, China) on UA-induced Min6 cell injury, Min6 cells were pretreated with various concentrations of AS-IV (0, 12.5, 25, and 50 *μ*mol/l) for 2 h followed by 5 mg/dl UA for another 24 h. To inhibit the PI3K/AKT pathway in Min6 cells, 100 nM of wortmannin (a PI3K/AKT pathway inhibitor; Sigma-Aldrich, St. Louis, MO, USA) was used.

### 2.3. MTT Assay

Min6 cell viability was evaluated using the MTT assay. Briefly, Min6 cells were treated with AS-IV, UA, or wortmannin. The cells were seeded in 96-well plates (10^4^ cells per well) and incubated for 24 h at 37°C. Thereafter, cells were treated with 10 *μ*l MTT (5 mg/ml; Sigma-Aldrich, St. Louis, MO, USA) and continuously incubated for 4 h. After removal of the MTT solution, 100 *μ*l of dimethyl sulfoxide (DMSO) was added to each well to solubilize the formazan product. After 15 min of vibration mixing, the optical density (OD) was measured at a wavelength of 570 nm using a microplate reader (Bio-Rad Laboratories, Hercules, CA, USA), according to the manufacturer's protocols.

### 2.4. Flow Cytometry Analysis

For apoptosis detection, Min6 cells were stimulated with AS-IV, UA, or wortmannin for the indicated time. Thereafter, the cells were washed, centrifuged, and resuspended. Cell apoptosis was evaluated using the annexin V-fluorescein isothiocyanate (FITC)/propidium iodide (PI) apoptosis detection kit (BD Biosciences, Franklin Lakes, NJ, USA) according to the manufacturer's instructions. Briefly, cells were incubated with 5 *μ*l annexin V-FITC and 5 *μ*l PI for 15 min at room temperature in the dark. A BD FACSCalibur flow cytometer (BD Biosciences, Franklin Lakes, NJ, USA) was employed to detect cell apoptosis (early apoptosis + late apoptosis). The flow cytometry results were analyzed using CellQuest software (version 5.1; BD Biosciences, Franklin Lakes, NJ, USA).

### 2.5. Glucose-Stimulated Insulin Secretion (GSIS) Assay

Min6 cells (5 × 10^4^ cells per well in 6-well plates) were cultured in DMEM at 37°C for 24 h. Thereafter, the cells were pretreated with various concentrations of AS-IV for 2 h followed by 5 mg/dl UA for another 24 h. The Krebs–Ringer Buffer (Sigma-Aldrich, St. Louis, Mo, USA) supplemented with 0.1% bovine serum albumin (BSA; Sigma-Aldrich, St. Louis, MO, USA) was applied to the cells, which were then incubated for 1 h. The cells were then stimulated with Krebs–Ringer Buffer containing low or high concentrations of glucose for another 1 h. Finally, the insulin content in samples was assessed by radioimmunoassay, as described previously.

### 2.6. Caspase-3 Activity Detection

Min6 cells were pretreated with AS-IV or/and wortmannin for 2 h followed by treatment with UA (5 mg/dl) for another 24 h. To determine the activity of caspase-3 in Min6 cells (2 × 10^7^ cells) from different groups, a caspase-3 activity detection kit (Beyotime, Shanghai, China) was used according to the manufacturer's instructions.

### 2.7. qRT-PCR Analysis

Min6 cells were exposed to AS-IV, UA, or wortmannin for the indicated time. TRIzol reagent (Invitrogen, Carlsbad, CA, USA) was used to extract total RNA from Min6 cells, according to the manufacturer's instructions. Total RNA was then reverse-transcribed into complementary (c) DNA with the Reverse Transcription Kit (Thermo, San Jose, CA, USA). The expression of AKT was detected using a SYBR Green PCR Master Mix Kit (TaKaRa, Japan). The primers for qPCR were obtained from Sangon Biotech (Shanghai, China). The reactions were run in triplicate on the ABI PRISM 7900 sequence detection system (Applied Biosystems, USA). Target gene expression was calculated using the 2^−ΔΔCt^ method.

### 2.8. Western Blot Analysis

Total protein was collected from the treated Min6 cells using a radioimmunoprecipitation assay (RIPA) lysis buffer (Beyotime, Shanghai, China). A Bicinchoninic Acid (BCA)™ Protein Assay Kit (Beijing Solarbio Science and Technology Co., Ltd., Beijing, China) was employed to determine protein concentration. Thereafter, the extracted protein samples (40 *μ*g per lane) were loaded and separated on a 10% sodium dodecyl sulfate-polyacrylamide gel electrophoresis (SDS-PAGE), and transferred onto a polyvinylidene fluoride (PVDF) membrane (EMD Millipore, Billerica, MA, USA). After blocking with 5% skimmed milk for 1 h at room temperature, the membranes were cultured at 4°C overnight with primary antibodies against p-AKT (cat. no. 4060; 1 : 1000 dilution; Cell Signaling Technology, Inc., Beverly, MA, USA), AKT (cat. no. 4685; 1 : 1000 dilution; Cell Signaling Technology, Inc., Beverly, MA, USA), or GAPDH (cat. no. 5174; 1 : 1000 dilution; Cell Signaling Technology, Inc., Beverly, MA, USA), respectively. After washing with phosphate-buffered saline (PBS)-0.1% Tween-20, the membranes were incubated with the secondary antibody (cat. no. 7074; 1 : 2000 dilution; Cell Signaling Technology, Inc., Beverly, MA, USA) for 2 h at room temperature. Finally, proteins were visualized using the enhanced chemiLuminescence (ECL) western blotting detection kits (BestBio, Shanghai, China) according to the manufacturer's protocol. The strips were evaluated by band intensity analysis using ImageJ software (version 1.46; National Institutes of Health, Bethesda, MD, USA).

### 2.9. Statistical Analysis

We used the Kolmogorov–Smirnov test to determine the normality of the data in SPSS. Statistical analysis was performed using Statistical Package for the Social Sciences (SPSS) 20.0 (IBM Corp.). All experiments were conducted more than 3 times. The data are expressed as mean ± standard deviation (SD) of three independent experiments. Differences between the two groups were estimated using an unpaired Student's *t*-test. Statistical differences among multiple groups were analyzed by one-way analysis of variance (ANOVA) followed by Tukey's post hoc tests. *P* < 0.05 was considered to indicate significant difference.

## 3. Results

### 3.1. Effects of AS-IV on Min6 Cell Cytotoxicity

**Figure 1:**
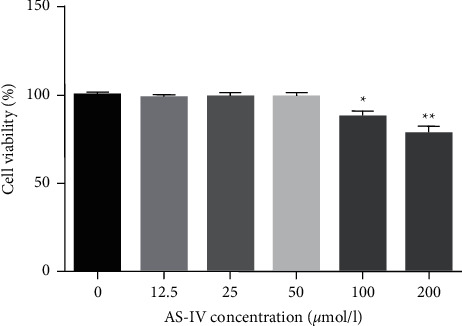
AS-IV had no obvious effect on Min6 cell viability. Min6 cells were stimulated with different concentrations of AS-IV. Then, Min6 cell viability was determined using the MTT assay. Data are expressed as mean ± SD. Statistical differences among groups were analyzed by one-way ANOVA followed by Tukey's post hoc tests. ^*∗*^,  ^*∗∗*^*P* < 0.05,  0.01 compared to the control group.

To determine whether AS-IV has functions in pancreatic *β*-cells, different concentrations of AS-IV (12.5, 25, 50, 100, and 200 *μ*mol/l) were applied to stimulate Min6 cells for 24 h. Supplementary Figure 1 shows the chemical formula of AS-IV. The MTT assay results revealed that 12.5, 25, and 50 *μ*mol/l of AS-IV did not influence the viability of Min6 cells (Figure 1), while 100 and 200 *μ*mol/l of AS-IV significantly reduced the viability of Min6 cells. Thus, 12.5, 25, and 50 *μ*mol/l AS-IV were selected for the following experiments.

### 3.2. Effects of AS-IV on UA-Stimulated Min6 Cell Viability and Apoptosis

**Figure 2:**
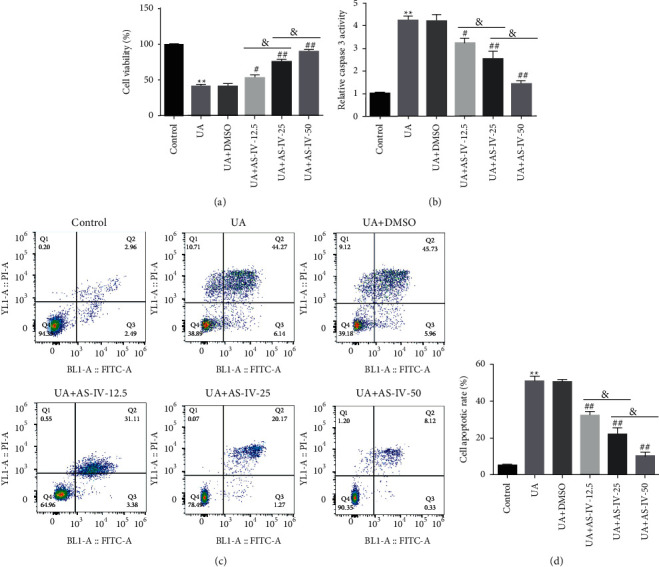
AS-IV abolished the influence of UA on Min6 cell viability and apoptosis. (a) Cell viability was evaluated by the MTT analysis. (b) Caspase-3 activity was determined in cells from the different groups. (c) Flow cytometry analysis was applied to quantify cell apoptosis. (d) Quantification of apoptotic cells. Data are expressed as mean ± SD. Statistical differences among groups were analyzed by one-way ANOVA followed by Tukey's post hoc tests. ^*∗∗*^*P* < 0.01 compared to the control group; ^#^,  ^##^*P* < 0.05,  0.01 vs. UA group; and *P* < 0.05.

To further illustrate the functions of AS-IV in UA-stimulated pancreatic *β*-cells, multiple concentrations of AS-IV (12.5, 25, and 50 *μ*mol/l) were used to pretreat Min6 cells for 2 h. Thereafter, Min6 cells were stimulated with 5 mg/dl UA for another 24 h. UA was found to prominently suppress Min6 cell viability (Figure 2(a)), enhance caspase-3 activity (Figure 2(b)), promote cell apoptosis, and markedly enhance the ratio of apoptotic cells (Figures 2(c) and 2(d)) in UA-treated Min6 cells. However, AS-IV abolished these results in a dose-dependent manner.

### 3.3. Effects of AS-IV on Insulin Secretion in Glucose-Stimulated Min6 Cells

**Figure 3:**
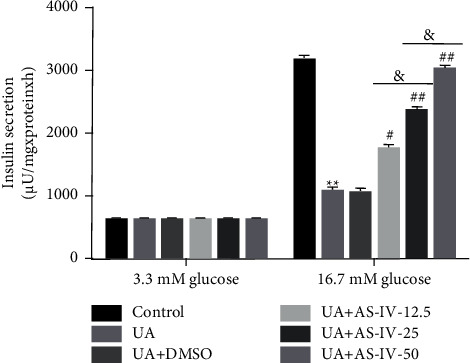
AS-IV promoted glucose-induced insulin secretion in Min6 cells. Min6 cells were stimulated with AS-IV, UA, and low or high concentrations of glucose. The cells were divided into six groups: control, UA, UA + DMSO, UA ± 12.5 *μ*mol/l AS-IV, UA + 25 *μ*mol/l AS-IV, and UA + 50 *μ*mol/l AS-IV groups. Insulin release was determined using the GSIS assay. Data are expressed as mean ± SD. Statistical differences among groups were analyzed by one-way ANOVA followed by Tukey's post hoc tests. ^*∗∗*^*P* < 0.01 compared to the control group; ^#^,  ^##^*P* < 0.05,  0.01 vs. UA group; and *P* < 0.05.

Then, this study revealed the roles of AS-IV in Min6 cell dysfunction and adopted AS-IV (12.5, 25, and 50 *μ*mol/l) and UA (5 mg/dl) to treat Min6 cells. Different concentrations of glucose were then used to stimulate Min6 cells for another 1 h. As displayed in Figure 3, UA remarkably reduced insulin secretion under 16.7 mM glucose treatment. Compared with UA, AS-IV evidently promoted high glucose-stimulated insulin production in a dose-dependent manner.

### 3.4. AS-IV Protects UA-Stimulated Cell Damage by Regulating the PI3K/AKT Signal Pathway

**Figure 4:**
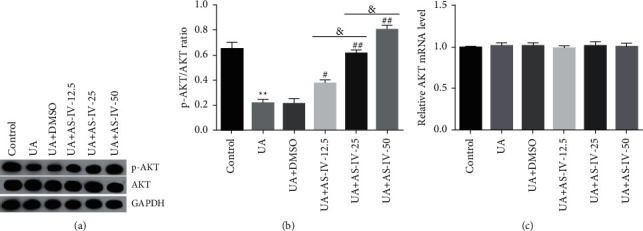
AS-IV activated the PI3K/AKT pathway in UA-treated Min6 cells. Min6 cells were stimulated with AS-IV or UA. The cells were divided into six groups: control, UA, UA + DMSO, UA + 12.5 *μ*mol/l AS-IV, UA ± 25 *μ*mol/l AS-IV, and UA ± 50 *μ*mol/l AS-IV groups. (a) Western blot analysis of p-AKT levels in the six groups. (b) The ratio of p-AKT/AKT. (c) mRNA levels of AKT were assessed using qRT-PCR. Data are expressed as mean ± SD. Statistical differences among groups were analyzed by one-way ANOVA followed by Tukey's post hoc tests. ^*∗∗*^*P* < 0.01 compared to the control group; ^#^,  ^##^*P* < 0.05,  0.01 vs. UA group; and *P* < 0.05.

To conduct an in-depth exploration of the related mechanism for the effect of AS-IV on UA-induced Min6 cells, the expression of proteins in the PI3K/AKT pathway was detected. Based on western blot and qRT-PCR, UA notably inhibited p-AKT protein levels and the ratio of p-AKT/AKT in Min6 cells. Nevertheless, after AS-IV treatment, the protein expression levels of p-AKT and the p-AKT/AKT ratio were amplified in a dose-dependent manner (Figures 4(a) and 4(b)). However, there were no significant changes in AKT mRNA levels among the different groups (Figure 4(c)).

### 3.5. Effect of Wortmannin on Pancreatic *β*-Cell Growth and Apoptosis in Min6 Cells after UA and AS-IV Treatment

**Figure 5:**
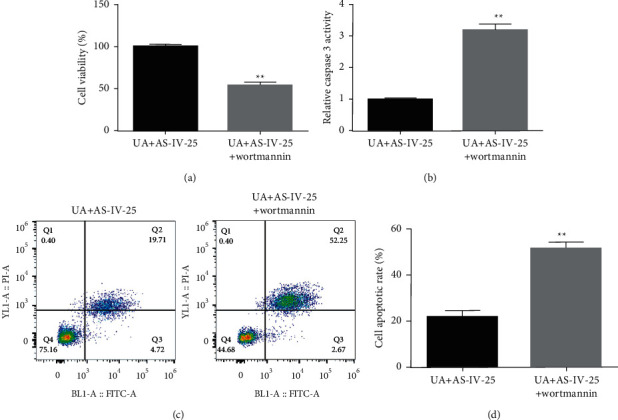
Wortmannin intercepted the protective effect of AS-IV in UA-stimulated Min6 cells. (a) Min6 cells were stimulated with UA and AS-IV/wortmannin. Cells were divided into two groups: UA ± 25 *μ*mol/l AS-IV and UA ± 25 *μ*mol/l AS-IV ± 100 nM wortmannin. (b) Caspase-3 activity was determined in cells from different groups. (c) Flow cytometry analysis was employed to quantify cell apoptosis. (d) Quantification of apoptotic cells. Data are expressed as mean ± SD. Differences between groups were estimated using the unpaired Student's *t*-test. ^*∗∗*^*P* < 0.01 vs. UA + AS-IV-25.

To further confirm whether AS-IV can resist UA-induced pancreatic *β*-cell damage and dysfunction by activating the PI3K/AKT pathway, wortmannin, a PI3K/AKT inhibitor, was applied. Min6 cells were treated with 25 *μ*mol/l AS-IV or/and 100 nM wortmannin for 2 h. Thereafter, the cells were treated with 5 mg/dl UA for another 24 h. As shown in Figure 5, 100 nM wortmannin obviously restrained Min6 cell viability (Figure 5(a)), enhanced caspase-3 activity (Figure 5(b)), and induced Min6 cell apoptosis (Figures 5(c) and 5(d)) when compared to the UA + 25 *μ*mol/l AS-IV group.

### 3.6. Effect of Wortmannin on Insulin Production in Min6 Cells after UA and AS-IV Treatment

**Figure 6:**
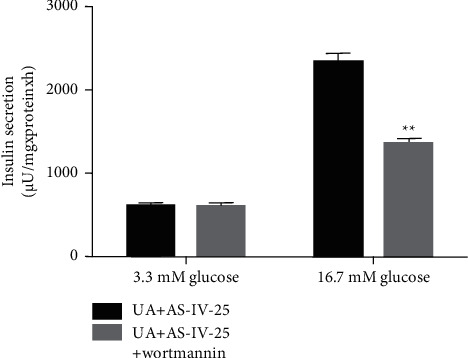
Wortmannin reduced the effect of AS-IV on insulin secretion in UA-stimulated Min6 cells. Min6 cells were stimulated with UA and AS-IV/wortmannin, as well as low or high concentrations of glucose. Cells were divided into two groups: UA + 25 *μ*mol/l AS-IV and UA ± 25 *μ*mol/l AS-IV ± 100 nM wortmannin. The production of insulin in six groups was calculated using the GSIS assay. Data are expressed as mean ± SD. Differences between groups were estimated using the unpaired Student's *t*-test. ^*∗∗*^*P* < 0.01 vs. UA + AS-IV-25.

As shown in Figure 6, 100 nM wortmannin markedly decreased insulin production in glucose-induced Min6 cells (Figure 6), suggesting that AS-IV exhibited protective effects on UA-induced Min6 cells via the PI3K/AKT pathway.

### 3.7. AS-IV Protects Pancreatic *β*-Cell Functions by Activating the PI3K/AKT Pathway in Min6 Cells after UA Treatment

**Figure 7:**
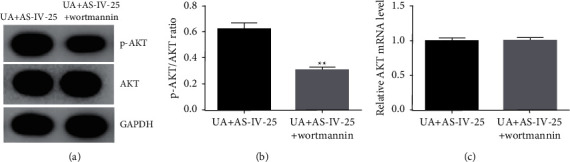
Wortmannin abolished the function of AS-IV in the PI3K/AKT pathway in UA-stimulated Min6 cells. Min6 cells were stimulated with UA and AS-IV/wortmannin. Cells were divided into two groups: UA ± 25 *μ*mol/l AS-IV and UA ± 25 *μ*mol/l AS-IV ± 100 nM wortmannin. (a) Western blot analysis of p-AKT levels in two groups. (b) The ratio of p-AKT/AKT. (c) mRNA levels of AKT were assessed using qRT-PCR. Data are expressed as mean ± SD. Differences between groups were estimated using the unpaired Student's *t*-test. ^*∗∗*^*P* < 0.01 vs. UA + AS-IV-25.

Finally, this study explored whether AS-IV protects pancreatic *β*-cell functions by activating the PI3K/AKT pathway in Min6 cells after UA treatment. Based on western blot and qRT-PCR, wortmannin significantly suppressed the protein expression of p-AKT (Figure 7(a)) and the ratio of p-AKT/AKT (Figure 7(b)) in Min6 cells after UA + 25 *μ*mol/l AS-IV stimulation. However, the mRNA levels of AKT were not significantly altered among various groups (Figure 7(c)).

## 4. Discussion

DM is a familiar chronic disease that leads to cell injury, increases serum UA, and accelerates the progression of DM [22]. T2D, which is a type of DM, is characterized by insulin disorder or *β*-cell injury [23]. In recent years, the potential relationship between UA level and the risk of T2D has been revealed in various clinical studies. For example, Guarda et al. revealed that high serum UA is associated with tubular damage and kidney inflammation in T2D patients [24]. Moreover, Wang et al. reported the relationship between serum UA and ischemic stroke in a large T2D population in China [25]. Currently, drug therapy is the main treatment for DM, including the dipeptidyl peptidase-4 inhibitor, sitagliptin, which is approved in more than 130 countries worldwide as monotherapy and in combination with other antihyperglycemic drugs for the treatment of adult patients with T2D. The pathogenesis of DM and the development of treatment strategies have always been a hot spot in this research field. AS-IV, an active saponin, is extracted from *Astragalus membranaceus*. A previous report revealed that AS-IV displayed various activities in diseases, including T2D [26]. Based on these assessments, this study opted to determine whether AS-IV could be a useful agent to relieve pancreatic *β*-cell dysfunction and evaluate the underlying mechanisms.

Pancreatic *β*-cell dysfunction is widely recognized as a significant feature of T2D [27]. Therefore, this investigation assessed the roles of AS-IV in pancreatic *β*-cells by employing different concentrations of AS-IV (12.5, 25, 50, 10, and 200 *μ*mol/l) to stimulate Min6 cells for 24 h. Firstly, the data showed that AS-IV had no cytotoxic effect on pancreatic *β*-cells. Based on previous studies, excess UA may induce insulin resistance and pancreatic *β*-cell injury, which was associated with the progression of T2D [7–13]. Lv et al. revealed that high serum UA may increase the risk of T2D [28]. The present study further explained the functions of AS-IV in UA-stimulated pancreatic *β*-cells. In partial accordance with other reports, this study revealed that UA evidently suppressed Min6 cell viability and promoted cell apoptosis compared to the control. Caspase-3 is a significant mediator of the apoptosis-related pathway [29]. Accordingly, caspase-3 activity was determined in this study. Based on the findings, caspase-3 activity was significantly enhanced in Min6 cells after UA stimulation. Nevertheless, UA-stimulated Min6 cell viability and apoptosis were abolished by AS-IV in a dose-dependent manner, suggesting that AS-IV alleviated pancreatic *β*-cell injury caused by UA.

Several reports have revealed that insulin resistance is the principal cause of T2D development. Owing to UA treatment, insulin secretion in pancreatic *β*-cells was obviously inhibited [30]. Hence, in the current study, insulin content in glucose-stimulated Min6 cells was determined. The results of the GSIS assay revealed that AS-IV significantly altered insulin production in a dose-dependent manner, indicating that AS-IV inhibited UA-induced Min6 cell dysfunction by suppressing apoptosis and stimulating insulin release.

Some pharmacological activities of AS-IV are closely related to the regulation of the PI3K/AKT signaling pathway [18–20]. The activation of the PI3K/AKT pathway has been reported to be responsible for the antiapoptotic effect of AS-IV [31]. Lu et al. revealed that AS-IV plays a protective role in chronic glomerulonephritis by activating autophagy through the PI3K/AKT/AS160 pathway [32]. To further verify our speculation, the PI3K/AKT pathway was determined. Findings revealed that the PI3K/AKT pathway was substantially inhibited in UA-treated Min6 cells. However, the effects of UA on the PI3K/AKT pathway were found to be reversed by AS-IV. These findings suggest that AS-IV prevented pancreatic *β*-cell from UA-induced damage by regulating the PI3K/AKT pathway. To further analyze the roles of the PI3K/AKT pathway in the regulation of pancreatic *β*-cell functions, the PI3K inhibitor wortmannin was employed. Wortmannin (100 nM) was found to remarkably suppress the protective effect of AS-IV on UA-treated Min6 cells, as demonstrated by decreased cell viability, insulin production, and enhanced apoptotic cells and caspase-3 activity. These findings further demonstrated that AS-IV protected pancreatic *β*-cell damage induced by UA by activating the PI3K/AKT pathway.

This study also had some limitations. For example, the effect of more doses of AS-IV on UA-treated Min6 cells was not assessed. Further, the effect of AS-IV on pancreatic *β*-cell function in T2D was not evaluated *in vivo*. These issues will be addressed in future studies.

In conclusion, this study revealed that AS-IV protected UA-induced pancreatic *β*-cell dysfunction by agitating the PI3K/AKT pathway. Altogether, the findings of the current study provided a better understanding of the potential mechanism of AS-IV on T2D and revealed that AS-IV may serve as a novel agent for the treatment of T2D.

## Data Availability

All datasets used and/or generated during the current study are available from the corresponding author on reasonable request.

## Supplementary Materials

Supplementary Materials Supplementary Figure 1 presents the chemical formula of AS-IV.
