# Supplementary figures and images for: Protective Effects of Astragaloside IV on Uric Acid-Induced Pancreatic β-Cell Injury through PI3K/AKT Pathway Activation
Source: Evid Based Complement Alternat Med. 2022 Jan 10;2022:2429162. doi: 10.1155/2022/2429162 (PMC8763508; doi:10.1155/2022/2429162)

Supplementary Figure 1. Chemical formula of AS-IV.

**
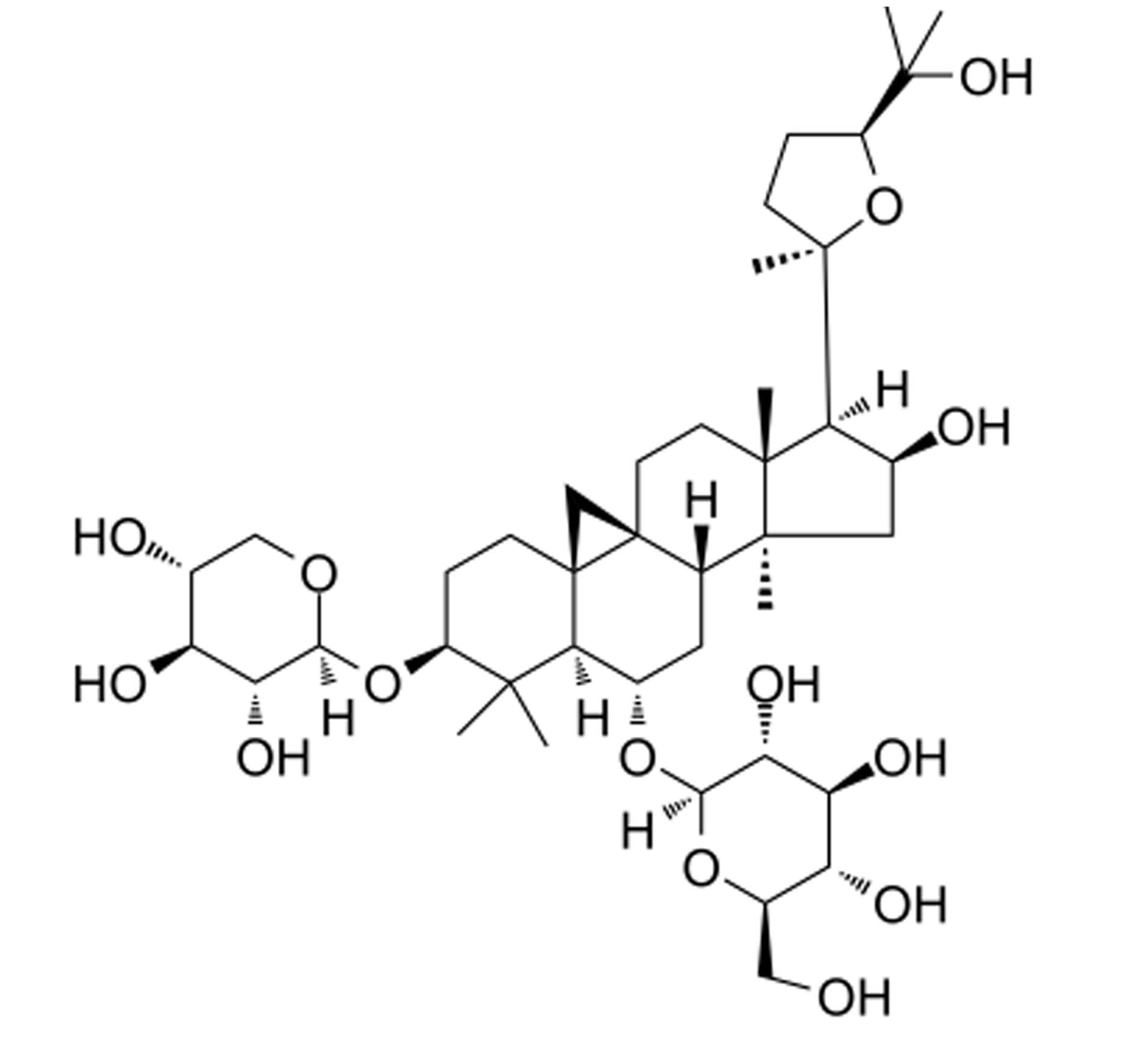
**

Supplement: Supplementary Materials — Supplementary Figure 1 presents the chemical formula of AS-IV. [file 2429162.f1.docx]
